# Case Report: Upper septal fascicular ventricular tachycardia mimicking supraventricular tachycardia: diagnostic pitfalls and mapping-guided ablation

**DOI:** 10.3389/fcvm.2026.1933819

**Published:** 2026-10-29

**Authors:** Yilin Fu, Jia Tang, Zixiang Zhao, Yongxiang Cai, Yang Chen

**Affiliations:** 1 School of Medicine, University of Electronic Science and Technology of China, Chengdu, China; 2 Department of Cardiology, Sichuan Provincial People’s Hospital, University of Electronic Science and Technology of China, Chengdu, China

**Keywords:** adenosine, bundle-branch block, cardiac electrophysiologic techniques, case report, catheter ablation, heart conduction system, supraventricular tachycardia, ventricular tachycardia

## Abstract

**Background:**

Upper septal fascicular ventricular tachycardia (VT) is a rare form of idiopathic left ventricular tachycardia arising from the proximal His-Purkinje system. Because near-synchronous ventricular activation can produce a relatively narrow QRS complex, this arrhythmia may closely mimic supraventricular tachycardia (SVT) with aberrant conduction.

**Case presentation:**

A 25-year-old man without structural heart disease presented with recurrent palpitations for more than 4 years. ECG during tachycardia showed a regular narrow-complex tachycardia at approximately 200 beats/min with atypical right bundle-branch block-like morphology and marked right-axis deviation. Electrophysiological study initially raised suspicion for an AVNRT-like mechanism because programmed atrial stimulation demonstrated dual AV nodal physiology and intracardiac recordings showed an apparent near-simultaneous ventriculoatrial relationship. However, 6 mg intravenous adenosine did not terminate the tachycardia but unmasked atrioventricular dissociation, with the ventricular rate exceeding the atrial rate; together with subsequent Purkinje-system mapping, this supported a ventricular origin. Initial mapping identified an effective target in the distal left posterior fascicular region, where 30-W radiofrequency ablation terminated the tachycardia. The same night, incessant tachycardia recurred with a changed and narrower QRS morphology. Repeat mapping localized the final successful target to the upper septal region approximately 8 mm from the His bundle; 20-W radiofrequency ablation terminated the tachycardia without atrioventricular conduction injury. No symptomatic recurrence was reported during 12-month clinical follow-up.

**Conclusion:**

A relatively narrow or progressively narrowing QRS complex does not exclude VT. In suspected SVT with atypical features, adenosine-unmasked atrioventricular dissociation should prompt reconsideration of a ventricular mechanism, while invasive electrophysiological assessment and repeat mapping may facilitate accurate diagnosis and safe ablation, particularly when the arrhythmogenic substrate is adjacent to the His bundle.

## Introduction

Regular narrow-QRS tachycardia is usually approached clinically as supraventricular tachycardia (SVT), especially in young patients without structural heart disease. However, a subset of idiopathic ventricular tachycardias (VTs) arising from the fascicular or proximal His-Purkinje system may present with a relatively narrow QRS complex, making the initial distinction from SVT difficult (1, 2).

Upper septal fascicular VT is uncommon and, because of its proximity to the proximal His-Purkinje system, may produce near-synchronous ventricular activation and a relatively narrow QRS complex, thereby mimicking SVT (3–5).

We report an upper septal fascicular VT that became incessant and further narrowed after initial ablation, highlighting the diagnostic value of adenosine-unmasked atrioventricular dissociation and repeat mapping.

The timeline of the patient's clinical course, diagnostic evaluation, interventions, and follow-up is summarized in Table 1.

**Table 1:** Timeline of the episode of care, including key clinical events, electrophysiological findings, ablation procedures, and follow-up.

Time point	Clinical event
>4 years before admission	Recurrent palpitations began, initially occurring approximately once or twice per year and lasting about 30 min.
>1 year before admission	Symptoms worsened and episode duration subjectively increased.
23 September 2025	Admission for recurrent palpitations. Routine laboratory tests and transthoracic echocardiography showed no significant abnormality or structural heart disease.
Initial tachycardia ECG	Regular narrow-complex tachycardia at approximately 200 beats/min with a QRS duration of 116 ms, rSR′/atypical RBBB morphology, and marked right-axis deviation.
24 September 2025	First electrophysiological study demonstrated the initial tachycardia (TCL, 288 ms), with dual AV nodal physiology, an AV nodal jump and echo beats, and near-simultaneous concentric VA activation, raising an initial working diagnosis of AVNRT. Slow-pathway ablation did not affect the tachycardia. Adenosine unmasked atrioventricular dissociation while the tachycardia persisted, supporting a ventricular origin.
Initial ablation	Activation mapping identified the earliest Purkinje-related activation in the distal left posterior fascicular region. Radiofrequency ablation using an irrigated contact-force catheter at 30 W terminated the tachycardia.
Night after first ablation	Incessant tachycardia recurred with a different morphology and further QRS narrowing from 116 ms to 97 ms. Biphasic 200-J cardioversion transiently terminated the tachycardia, but rapid recurrence occurred. Intravenous propafenone, lidocaine, amiodarone, and diltiazem failed to maintain sinus rhythm, prompting an early repeat electrophysiological study and catheter ablation the following day.
25 September 2025	Repeat electrophysiological study induced VT2 (TCL, 354 ms) with a changed and narrower QRS morphology. Repeat mapping identified the earliest Purkinje-related activation in the upper septal region. The final effective ablation site was approximately 8 mm from the His recording site.
Second ablation	Careful low-power radiofrequency ablation at the upper septal target (20 W) terminated the tachycardia. No clinically evident atrioventricular conduction disturbance occurred during or after ablation.
Follow-up	No symptomatic recurrence was reported during 12-month clinical follow-up.

VT, ventricular tachycardia; ECG, electrocardiogram; RBBB, right bundle-branch block; AVNRT, atrioventricular nodal reentrant tachycardia; VA, ventriculoatrial; TCL, tachycardia cycle length.

## Case description

### Patient information and clinical findings

A 25-year-old man presented with recurrent palpitations for more than 4 years, worsening over the preceding year. Episodes initially occurred once or twice annually, lasted approximately 30 min, and were accompanied by exertional fatigue or chest discomfort. SVT had previously been suspected at an outside facility, although the exact rhythm diagnosis remained unclear.

He had no relevant past medical history. Routine laboratory tests, including blood count and biochemical testing, revealed no clinically significant abnormalities. Transthoracic echocardiography showed no structural heart disease and preserved cardiac function, with a left ventricular ejection fraction of 62%.

### Initial diagnostic assessment

During a symptomatic episode, 12-lead electrocardiography (ECG) showed a regular narrow-complex tachycardia at approximately 200 beats/min with a QRS duration of 116 ms. Lead V1 showed an rSR′/atypical right bundle-branch block (RBBB) morphology, the inferior leads showed an rS pattern, and the frontal axis was markedly rightward (Figure 1A). The relatively narrow QRS complex, young age, and absence of structural heart disease initially suggested SVT with aberrancy; however, the atypical RBBB-like morphology and marked right-axis deviation prompted consideration of AVNRT, AVRT, atrial tachycardia, and fascicular VT.

**Figure 1:**
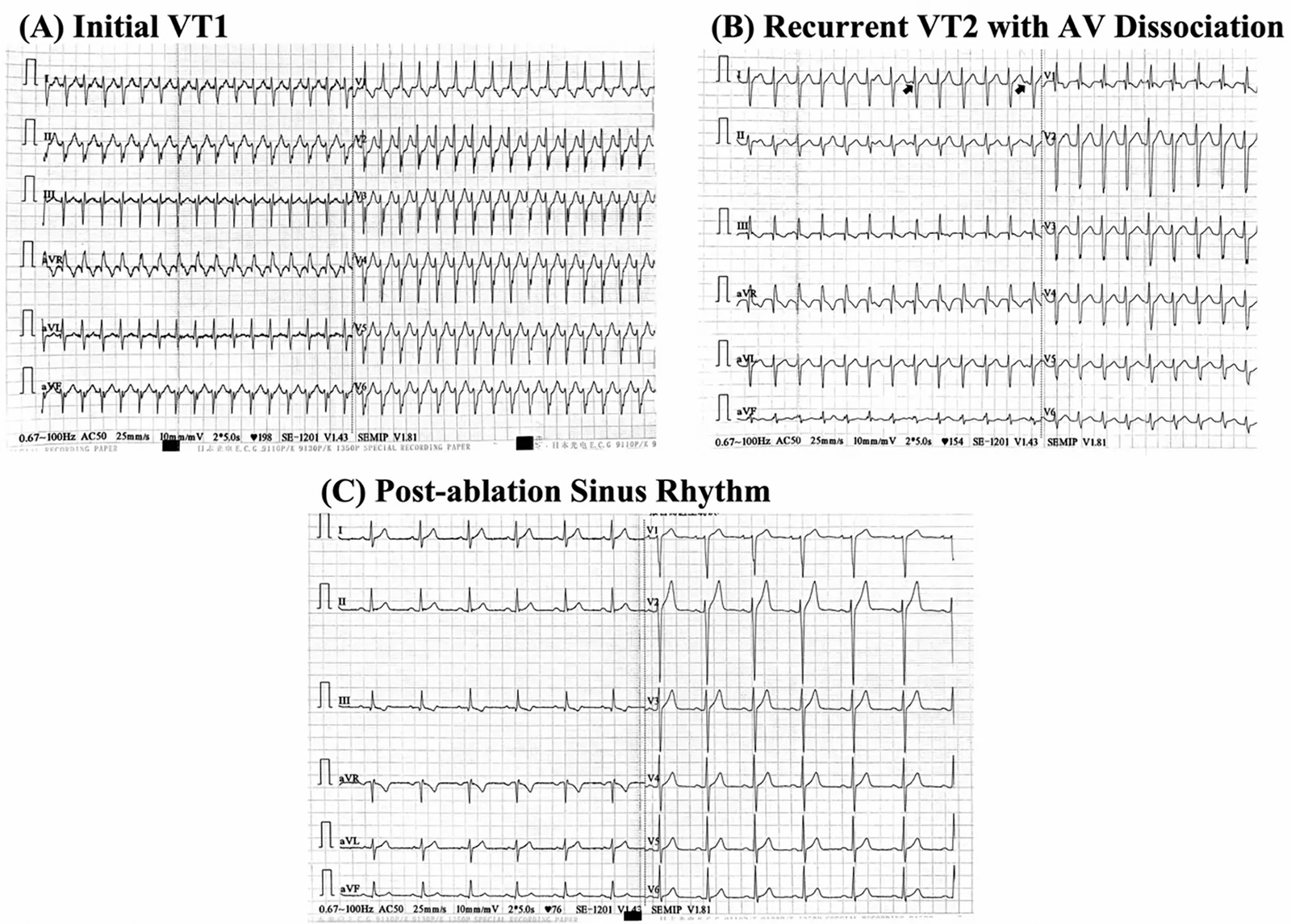
Surface electrocardiographic findings during the clinical course. **(A)** Preablation 12-lead electrocardiogram obtained during the initial tachycardia, demonstrating a narrow-QRS tachycardia with a QRS duration of 116 ms, an rSR′ morphology in lead V1, and marked right-axis deviation. **(B)** Electrocardiogram obtained during recurrence after the initial ablation, demonstrating incessant tachycardia with a changed morphology and further QRS narrowing from 116 ms to 97 ms. Black arrows indicate dissociated atrial activity. **(C)** Postprocedural 12-lead electrocardiogram showing sinus rhythm without evidence of clinically significant atrioventricular conduction block.

### First electrophysiological study and initial ablation

The following day, an electrophysiological study and radiofrequency catheter ablation were performed. A coronary sinus catheter was positioned, and a decapolar mapping/navigation catheter (DecaNav) was used for ventricular mapping. Tachycardia was induced using isoproterenol infusion and programmed stimulation. During baseline sinus rhythm, the AH and HV intervals were 81 ms and 46 ms, respectively, with an antegrade A–H–P–V activation sequence.

During programmed atrial stimulation, shortening the S1S2 coupling interval from 500/360 ms to 500/350 ms produced an approximately 92-ms AV nodal jump, and AV nodal echo beats were observed, supporting dual AV nodal physiology. During the initial tachycardia, with a tachycardia cycle length (TCL) of 288 ms, coronary sinus recordings demonstrated a concentric atrial activation sequence with near-simultaneous ventriculoatrial activation (Figure 2A). Available His-bundle recordings during the initial tachycardia showed an HV interval of 26 ms, with Purkinje/ventricular activation preceding retrograde His activation. Ventricular pacing could induce or terminate the tachycardia, making an atrial tachycardia mechanism less likely. Ventricular entrainment during the initial tachycardia was attempted but could not be successfully achieved. Atrial pacing during the initial tachycardia did not produce clear resetting of the tachycardia. In addition, a ventricular extrastimulus delivered during tachycardia did not alter the VV interval. During sinus rhythm, ventricular pacing demonstrated decremental ventriculoatrial conduction with Wenckebach behavior, arguing against a retrogradely conducting accessory pathway. Together with the relatively narrow QRS morphology and a pseudo-S-wave appearance in lead II, these findings made AVNRT a plausible initial working diagnosis. Electroanatomical mapping and catheter navigation were performed using the CARTO 3 system. Although dual AV nodal physiology alone was not diagnostic of AVNRT, slow-pathway ablation was attempted on the basis of the overall electrophysiological findings in temperature-controlled mode at 30 W for 180 s. However, the ablation did not modify the tachycardia, which persisted and remained recurrent. Because the mechanism remained uncertain after the ineffective slow-pathway ablation attempt, 6 mg intravenous adenosine was administered to transiently suppress atrioventricular nodal conduction and assess the tachycardia response for further differential diagnosis. The tachycardia persisted, with no appreciable change in TCL, ventricular rate, or surface QRS morphology; however, atrioventricular dissociation became evident, with the ventricular rate exceeding the atrial rate (Figure 2B). This adenosine-unmasked atrioventricular dissociation strongly argued against AVRT and an atrial-driven tachycardia and made the initially suspected AVNRT mechanism less likely. Together with the subsequent Purkinje-related mapping findings, it supported a ventricular origin.

**Figure 2:**
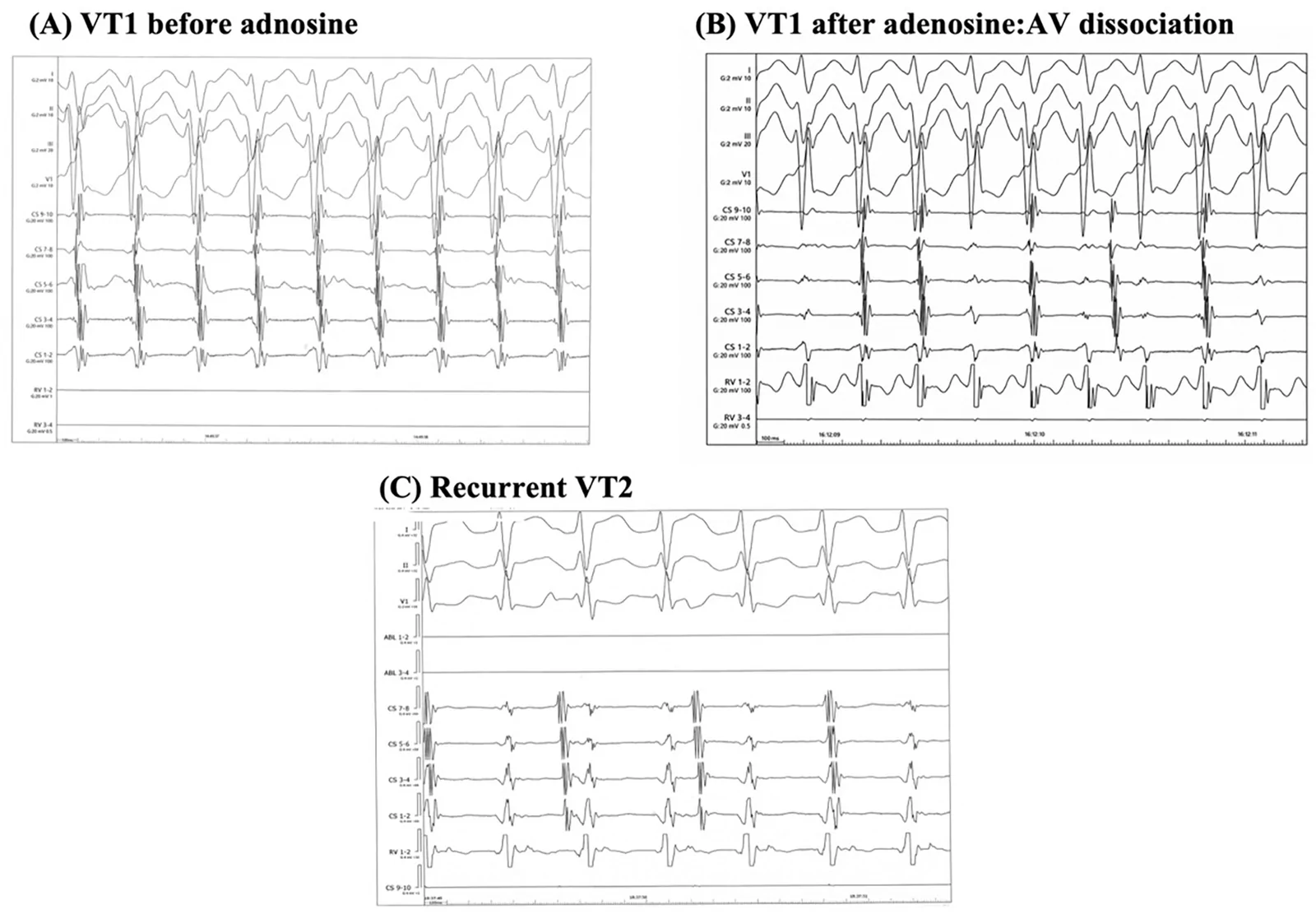
Intracardiac electrophysiological findings illustrating the diagnostic evolution. **(A)** During the initial ventricular tachycardia (VT1; TCL, 288 ms), intracardiac recordings demonstrated a concentric atrial activation sequence with near-simultaneous ventriculoatrial activation, contributing to the initial suspicion of an AVNRT-like mechanism. **(B)** Following intravenous administration of 6 mg adenosine, the tachycardia persisted without appreciable change in cycle length, ventricular rate, or surface QRS morphology, while atrioventricular dissociation became evident with the ventricular rate exceeding the atrial rate. **(C)** Intracardiac recording of the recurrent ventricular tachycardia (VT2; TCL, 354 ms), corresponding to a ventricular rate of approximately 170 beats/min, before repeat mapping and ablation.

Subsequent activation mapping identified the earliest Purkinje-related activation in the distal left posterior fascicular region (Figure 3A), where a discrete Purkinje potential with a local P–V interval of approximately 34 ms was recorded. Ablation was performed using a THERMOCOOL SMARTTOUCH catheter at 30 W, with an irrigation flow rate of 15 mL/min and a contact force of 5 g. The tachycardia terminated after 5 s, followed by three 30-second consolidation lesions. After 30 min of observation and repeated isoproterenol infusion, no ventricular premature beats or VT were induced, and the procedure was ended.

**Figure 3:**
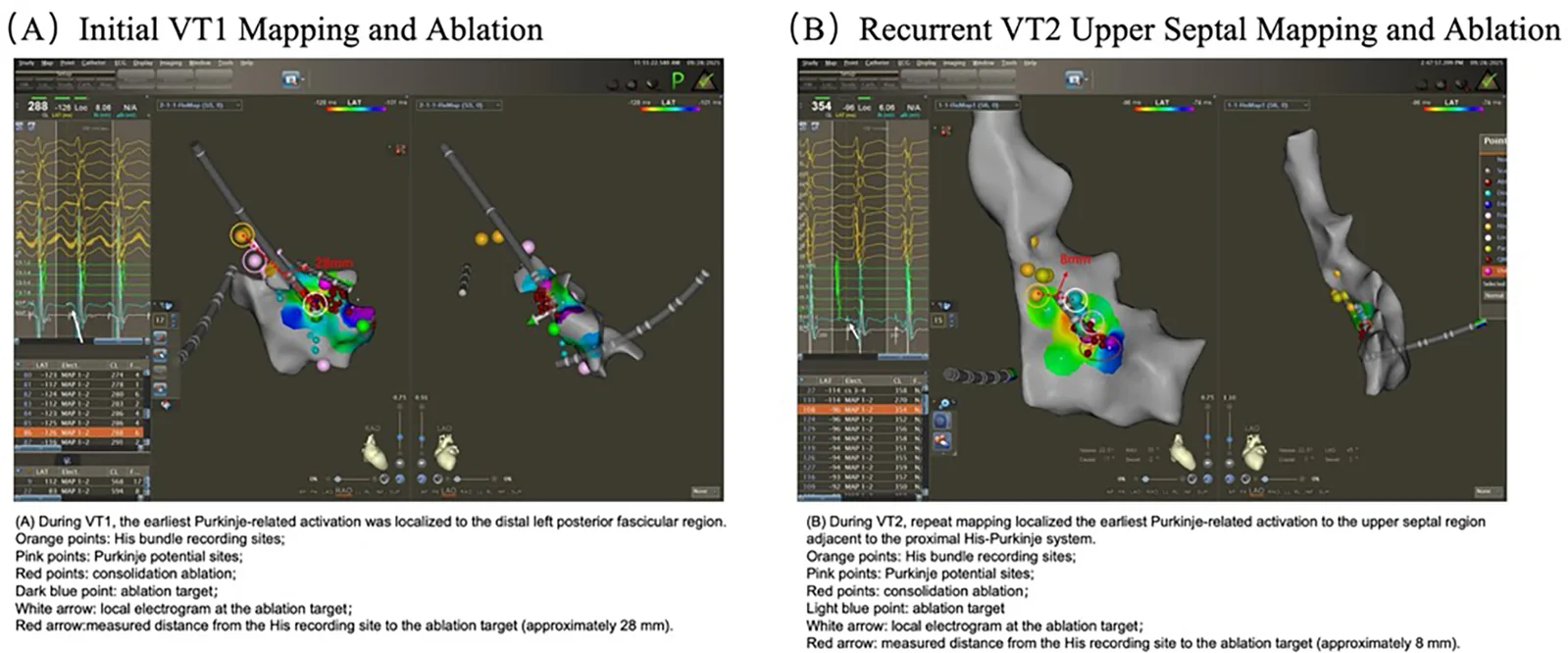
Electroanatomical mapping and sequential ablation sites. **(A)** Electroanatomical mapping during the initial ventricular tachycardia (VT1) localized the earliest Purkinje-related activation to the distal left posterior fascicular region, with a system-calculated local activation time (LAT) of −126 ms relative to the surface QRS reference (CARTO 3 V7 Advanced Reference Annotation). Orange markers indicate His bundle recording sites. The Purkinje potential and the first effective ablation site are indicated separately. The white arrow indicates the local electrogram recorded at the effective ablation site, and the red arrow indicates the measured distance from the His recording site to the effective ablation site (approximately 28 mm). **(B)** Repeat electroanatomical mapping during the recurrent ventricular tachycardia (VT2) localized the earliest Purkinje-related activation to the upper septal region adjacent to the proximal His-Purkinje system, with a system-calculated local activation time (LAT) of −96 ms relative to the surface QRS reference (CARTO 3 V7 Advanced Reference Annotation). Orange markers indicate His bundle recording sites. The Purkinje potential and the final effective ablation site are indicated separately, with additional consolidation lesions shown around the target region. The white arrow indicates the local electrogram recorded at the effective ablation site, and the red arrow indicates the approximately 8-mm distance from the His recording site to the final effective ablation site.

### Recurrence and repeat mapping

The same night, tachycardia recurred and became incessant. Repeat ECG showed incessant tachycardia at approximately 170 beats/min with a morphology different from the initial presentation and further QRS narrowing from 116 ms to 97 ms (Figure 1B). Lead V1 showed a changed RBBB-like pattern, the inferior leads suggested a left anterior fascicular block-like morphology, and the axis remained rightward (Figure 1B). Biphasic direct-current cardioversion at 200 J transiently terminated the tachycardia for only several seconds, followed by rapid recurrence. Intravenous propafenone, lidocaine, amiodarone, and diltiazem were ineffective in maintaining sinus rhythm. Verapamil was not administered because it was unavailable at the time. Given the incessant recurrence with symptomatic hypotension, only transient response to cardioversion, and failure of multiple intravenous antiarrhythmic agents, an early repeat electrophysiological study and catheter ablation were considered clinically necessary and were performed the following day. Ventricular programmed stimulation induced the recurrent tachycardia (VT2), with a TCL of 354 ms and a changed, narrower QRS morphology. Available His-bundle recordings during VT2 showed an HV interval of 15 ms, with Purkinje/ventricular activation preceding retrograde His activation. Repeat electroanatomical mapping identified the earliest Purkinje-related activation in the upper septal region adjacent to the proximal His-Purkinje system. At the final effective ablation site, the local Purkinje-to-ventricular (P–V) interval was approximately 45 ms. The final effective ablation site was located approximately 8 mm from the His recording site (Figure 3B), favoring an upper septal fascicular origin rather than an isolated typical left posterior fascicular VT. Because of the proximity to the His bundle, a cautious low-power ablation strategy was adopted. Atrioventricular conduction was closely monitored during radiofrequency delivery, with immediate cessation of energy planned in the event of abnormal junctional rhythm, AV conduction prolongation, or atrioventricular block. Radiofrequency energy was delivered at 20 W with an irrigation flow rate of 8 mL/min and a contact force of 5 g. The tachycardia terminated after 5 s, followed by three 30-second consolidation lesions. No clinically evident atrioventricular conduction disturbance occurred during or after ablation.

### Follow-up and outcomes

Post-ablation ECG showed sinus rhythm without clinically significant atrioventricular conduction disturbance (Figure 1C). No symptomatic recurrence was reported during 12-month clinical follow-up, although systematic ambulatory monitoring was not performed.

## Discussion

This case illustrates the diagnostic challenge posed by Purkinje-related ventricular tachycardia presenting with a relatively narrow QRS complex and AVNRT-like electrophysiological findings (1, 2). Although dual AV nodal physiology, AV nodal echo beats, and near-simultaneous concentric ventriculoatrial activation initially raised suspicion for AVNRT, adenosine-unmasked atrioventricular dissociation during persistent tachycardia redirected the diagnosis toward a ventricular mechanism.

Upper septal fascicular VT, also referred to as upper septal idiopathic left ventricular tachycardia (US-ILVT), is a rare subtype of idiopathic fascicular VT (3, 5). Fascicular VT is generally considered a Purkinje-related reentrant tachycardia (1). An upper septal origin near the proximal His-Purkinje network may permit rapid propagation through both bundle branches, resulting in near-synchronous ventricular activation and a relatively narrow QRS complex (3–5). This explains why US-ILVT can mimic SVT with aberrant conduction, particularly when coincidental findings such as dual AV nodal physiology are present. Our case was notable for two narrow-QRS tachycardias with different morphologies and sequentially different effective ablation sites.

The relationship between the initial and recurrent tachycardias remains uncertain. Possible explanations include two distinct but anatomically related fascicular VTs, different exits from a common Purkinje-related reentrant circuit, or modification of a single reentrant pathway following the initial ablation. In the absence of comprehensive entrainment or resetting data, the precise mechanistic relationship between the two tachycardias cannot be definitively established (6). Regardless of the precise mechanism, the change in QRS morphology and mapping findings underscores the value of repeat activation mapping when tachycardia recurs after an initially successful ablation (7). Although studied in a different substrate, recent work using strategically targeted multielectrode mapping in hemodynamically unstable scar-related VT further supports the value of focused high-resolution mapping for rapidly identifying critical VT pathways when conventional activation mapping is challenging (8).

Ablation near the His bundle requires a careful balance between efficacy and safety (4, 5). In this case, low-power radiofrequency delivery with continuous monitoring of atrioventricular conduction allowed successful ablation without clinically evident conduction injury.

### Limitations

This case has several limitations, including its single-patient design, incomplete documentation of antiarrhythmic drug dosing and timing, absence of systematic ambulatory rhythm monitoring, and incomplete systematic electrophysiological recordings. These limitations preclude definitive characterization of the tachycardia circuit and complete exclusion of asymptomatic recurrence.

### Take-home messages

This case highlights an important diagnostic pitfall: a relatively narrow QRS complex does not exclude VT, particularly when the QRS morphology or axis is atypical. Purkinje-related VT may mimic SVT through rapid His-Purkinje activation, while coincidental dual AV nodal physiology and near-simultaneous ventriculoatrial activation may further suggest AVNRT. When the mechanism remains uncertain, persistence of tachycardia with unchanged ventricular rate and QRS morphology despite adenosine-unmasked atrioventricular dissociation should prompt reconsideration of a ventricular origin. Careful His-Purkinje assessment and repeat activation mapping are therefore important, particularly when recurrence is accompanied by a changed or even narrower QRS morphology.

## Patient perspective

The patient provided written informed consent for publication of this case report and the accompanying images. No separate patient narrative statement was available.

## Data Availability

The datasets presented in this article are not readily available because additional patient-level raw data are not publicly available due to patient privacy, confidentiality, and ethical restrictions. The relevant data supporting the conclusions of this article are included in the manuscript. Potentially identifiable patient information will not be shared. Requests to access the datasets should be directed to YChen (chenyang@med.uestc.edu.cn).
